# Comparing Facial Attractiveness and Professional Acceptance Between Real and AI‐Generated Orthodontic Treatment Outcome Images

**DOI:** 10.1111/ocr.70144

**Published:** 2026-05-18

**Authors:** Gil Guilherme Gasparello, Otso Tirkkonen, Sergio Luiz Mota‐Júnior, Felipe Calgaro Martins, Marco Antonio Dias da Silva, Orlando Motohiro Tanaka

**Affiliations:** ^1^ Pontifícia Universidade Católica Do Paraná – PUCPR School of Medicine and Life Sciences, Dentistry Department Curitiba PR Brazil; ^2^ Research Unit of Population Health University of Oulu Oulu Finland; ^3^ The Wellbeing Services County of North Ostrobothnia Oulu Finland; ^4^ Universidade Federal do Rio de Janeiro Rio de Janeiro Brazil; ^5^ Research Group of Teleducation and Teledentistry Federal University of Campina Grande Campina Grande Brazil

**Keywords:** artificial intelligence, digital aesthetics, employability, facial attractiveness, orthodontics, perception AI‐generated images

## Abstract

**Background:**

Facial attractiveness influences social and professional perceptions, including competence and employability. With the increasing use of artificial intelligence (AI) to generate photorealistic faces, it is important to understand how these images compare to real orthodontic treatment outcomes.

**Objective:**

To compare perceptions of real versus AI‐generated orthodontic treatment results in terms of attractiveness, employability, and distinguishability.

**Methods:**

A cross‐sectional online survey included 288 Brazilian adults.

**Participants:**

Dentists (*n* = 76), dental students (*n* = 63), and laypeople (*n* = 149). Each evaluated three sets of images (pre‐treatment, post‐treatment, and AI‐generated post‐treatment) for three patients. Participants rated facial attractiveness (VAS 0–100), assessed employability, and identified whether the image was AI‐generated.

**Results:**

AI‐generated images were rated significantly more attractive than real post‐treatment ones (mean VAS: AI = 68.8; Real = 54.4; *p* < 0.001). Dentists, students, and laypeople all rated AI images higher. Employability was also greater for AI images (50.4%) than real ones (39.1%) (*p* = 0.007). Misclassification was common, with dental students achieving the highest accuracy (66.8%).

**Conclusion:**

Participants showed limited ability to accurately distinguish between real and AI‐generated images. AI‐generated images were perceived as more attractive and had a greater positive impact on employability judgements compared to real post‐treatment images.

## 1 Introduction

Facial and smile attractiveness play a central role in human social interactions, significantly influencing perceptions of personality, competence, and employability [1]. Orthodontic treatment is fundamentally connected to these aesthetic components, as it aims not only to establish functional occlusion but also to optimize smile aesthetics and overall facial harmony [2]. Consequently, the enhancement of facial attractiveness is frequently a primary motivation for patients seeking orthodontic care, and the success of such treatments is often judged by the resulting aesthetic improvements [1].

Facial attractiveness plays a significant role in both social and professional contexts. It is often associated with perceptions of competence, trustworthiness, and employability [1, 2]. Orthodontic treatment, beyond its functional benefits, can enhance facial aesthetics and potentially influence how individuals are perceived by others [3]. Simultaneously, the rapid advancement of artificial intelligence (AI) has introduced powerful tools for generating highly realistic synthetic facial images, which can simulate various aesthetic patterns [4], including appearances after aesthetic medical treatment outcomes.

These AI‐driven technologies, commonly referred to as “deep fakes,” have democratized access to sophisticated audiovisual synthesis techniques that were once exclusive to major film studios. Such technologies are capable of generating speech in any individual's voice [5], producing photorealistic synthetic faces [6], and modifying or swapping identities in video content [7]. While these innovations offer creative and entertainment possibilities, they also present serious ethical and societal challenges, including deception, manipulation, and the erosion of trust in visual media.

An additional clinical concern relates to the potential discrepancy between AI‐generated facial modifications and biologically achievable orthodontic outcomes [8]. Artificial intelligence systems may generate aesthetically optimized smiles that do not necessarily respect anatomical, skeletal, periodontal, or functional limitations [8]. Such digitally idealized representations may contribute to unrealistic patient expectations, particularly in an era where social media and digital self‐presentation already amplify aesthetic standards [9]. Understanding whether these synthetic outcomes are perceived as superior to real treatment results is therefore clinically relevant, as it may influence how patients evaluate orthodontic success and form expectations prior to treatment.

A core technology behind these capabilities is the Generative Adversarial Network (GAN), which consists of two competing neural networks: a generator and a discriminator. The generator attempts to produce increasingly realistic images, such as human faces, starting from random noise, while the discriminator tries to distinguish between real and synthetic images. Through iterative refinement, the generator learns to create faces that are virtually indistinguishable from real photographs [6]. The misuse of deep fakes has raised widespread concern. They have been used to create nonconsensual explicit content, perpetrate fraud, and fuel misinformation. Perhaps most troubling is the fact that, in a digital environment where any image or video can be fabricated, the authenticity of even genuine materials can be questioned [4].

This creates a “liar's dividend”, a situation in which the existence of deep fakes can be used to deny the reality of inconvenient truths. Although several detection methods have been developed to identify AI‐manipulated media [10, 11, 12], current tools lack the accuracy and scalability required to manage the immense volume of digital content produced daily [13]. As a result, the responsibility of distinguishing real from fake content increasingly falls on everyday users navigating an oversaturated online environment. Hence, this study aims to investigate public perceptions of facial attractiveness and professional appeal by comparing Real Facial Orthodontic Treatment Outcome (RFO) and AI Generated Facial Orthodontic Treatment Outcomes (AIGFO). Understanding how synthetic faces are evaluated relative to real ones has important implications for aesthetic dentistry, patient communication, and the management of treatment expectations.

## 2 Objective

Our objective was to compare the perception of AIGFO and RFO in terms of distinguishability, facial attractiveness, and professional acceptance. Furthermore, our objective was also to compare opinions of laypeople, dentists, and dental students. The study hypotheses were:

*AI‐generated orthodontic treatment outcome images (AIGFO) would be perceived as more attractive than real orthodontic treatment outcomes (RFO)*.

*Participants with dental training would demonstrate greater ability to distinguish AI‐generated from real images*.

*AI‐generated images would be associated with higher perceived professional acceptance compared to real treatment outcomes*.

## 3 Material and Methods

### 3.1 Study Design

This cross‐sectional observational study conducted through an anonymous online survey, with all responses provided voluntarily. No personal data or identifying information were collected, and participants were fully informed about the purpose of the research before providing their consent, following recognized ethical standards for anonymous online research. Additionally, written permission was obtained from all individuals whose facial photographs were included, authorizing the use of their anonymized images for research and academic dissemination. This study was approved by the Research Ethics Committee of the Pontifícia Universidade Católica do Paraná, Brazil, under approval number 6.861.105, CAAE: 77741824.5.0000.0020. The study was conducted in full accordance with the principles of the Declaration of Helsinki. All participants provided informed consent prior to participation, and the study was conducted in accordance with the Declaration of Helsinki and relevant institutional guidelines. Participation was voluntary, anonymous, and devoid of identifiable data.

The research question has now been structured according to the PICO framework: Population (P): Adults residing in Brazil (dentists, dental students, and laypeople). Intervention (I): Exposure to AI‐generated orthodontic treatment outcome images (AIGFO). Comparison (C): Real orthodontic treatment outcome images (RFO). Outcome (O): Perceived attractiveness, professional acceptance, and ability to distinguish AI‐generated from real images.

### 3.2 Image Selection and Processing

Three anonymized full‐face pre‐treatment photographs were selected from a personal clinical archive. The selection process was conducted independently by a panel of five experienced orthodontic specialists (≥ 5 years of clinical experience). Each specialist independently selected two cases from the pool that they considered representative of common orthodontic clinical presentations. These independently selected cases were subsequently discussed collectively, and through consensus, three final cases were chosen for inclusion in the study. The selection criteria included image quality, standardized photographic documentation, and clear presentation of distinct clinical scenarios. The purpose of this selection process was to identify clinically representative treatment situations.

1. A male patient (23.0 years at the beginning of treatment; 25.0 years at treatment completion) presenting with anterior open bite.
2. A female patient (21.0 years at the beginning of treatment; 22.9 years at treatment completion) presenting with dental crowding.
3. A female patient (20.4 years at the beginning of treatment; 22.3 years at treatment completion) who underwent orthognathic surgery as part of a surgery‐first treatment plan. (See Supporting Information S1).

For each selected case, three image types were prepared:

- The original pre‐treatment smiling full‐face frontal photograph
- A post‐treatment photograph following orthodontic or surgical intervention.
- An AI‐generated image was created using the paid version of ChatGPT (Version GPT‐4o, OpenAI, San Francisco, United States) on April 19, 2025. A newly registered account was used exclusively for image generation. Each of the pre‐treatment images was individually uploaded into the system to guide the generation process, using the only single prompt: “Create a perfect and realistic smile”.

The AI‐generated images were produced using the pre‐treatment frontal smiling photographs as input. The real post‐treatment photographs were obtained immediately after completion of orthodontic or surgical treatment under standardized clinical photographic conditions. The time interval between pre‐treatment and post‐treatment photographs corresponded to the duration of treatment (approximately 2 years for each case). No additional time‐related image modifications were applied.

Only standardized full‐face frontal smiling photographs were included in the study. No lateral or resting‐position images were used. All photographs were captured under standardized clinical conditions using professional photographic equipment, ensuring consistency in lighting, head positioning, framing, and facial expression.

### 3.3 Sample Size Estimation

The required sample size was initially calculated for a cross‐sectional proportion estimate using a 95% confidence level, a 6% margin of error (±6 percentage points around the estimated proportion), and an assumed response proportion of 50%, which maximizes variance. This approach yielded a minimum required sample of 267 participants. Additionally, to ensure adequate statistical power for comparisons between AI‐generated and real image classifications, a power analysis for two independent proportions was conducted. Assuming an increase in correct identifications from 50% to 75% (Δ = 0.25; Cohen's h ≈0.52), with two‐sided α = 0.05, power = 0.80, and equal allocation, the required total sample size was estimated at 116 participants (58 per group). The final sample of 288 participants exceeded both requirements, ensuring adequate precision of proportion estimates and sufficient statistical power for inferential comparisons.

### 3.4 Participants

Participants were recruited through professional networks, dental schools, and social media channels to ensure diversity in background and familiarity with dental and facial aesthetics. The final sample included three groups:

- Practicing dentists (*n* = 76),
- Undergraduate dental students (*n* = 63),
- Laypeople without formal dental training (*n* = 149).

Eligibility was restricted to individuals residing in Brazil and aged 18 years or older.

### 3.5 Data Collection Procedure

Data collection was conducted via the Qualtrics online survey platform over a two‐week period between 26th of April and 8th of May of 2025. The survey was self‐administered and completed individually using personal electronic devices (desktop, laptop, tablet, or smartphone).

Participants were presented with three images per patient (pre‐treatment, post‐treatment, and AI‐generated), totaling nine images. The order of image presentation was fully randomized for each participant using the Qualtrics randomization function to minimize order effects and reduce response bias. The sequence of cases and image types was independently randomized to prevent pattern recognition. No fixed viewing time was imposed for each photograph, allowing participants to observe each image for as long as they deemed necessary before responding. Similarly, no time restriction was placed on answering the questions. This approach was chosen to simulate naturalistic image evaluation conditions and avoid artificial time pressure that could influence perceptual judgement.

For each image, participants answered three questions:

1. **Image Source Classification:**“Do you believe this image was created by Artificial Intelligence?” (Response options: Yes/No)
2. **Attractiveness Assessment:**“On a scale from 0 to 100, how attractive do you find this person's facial appearance?” (0 = Not attractive at all; 100 = Extremely attractive)
3. **Employability Judgement:**“Would you consider hiring this person for a professional position?” (Response options: Yes/No/Not possible to determine based on image alone)

Participants were required to provide a response to each question before proceeding to the next image, ensuring completeness of image‐level data. The average total survey completion time was approximately 4 min.

**FIGURE 1:**
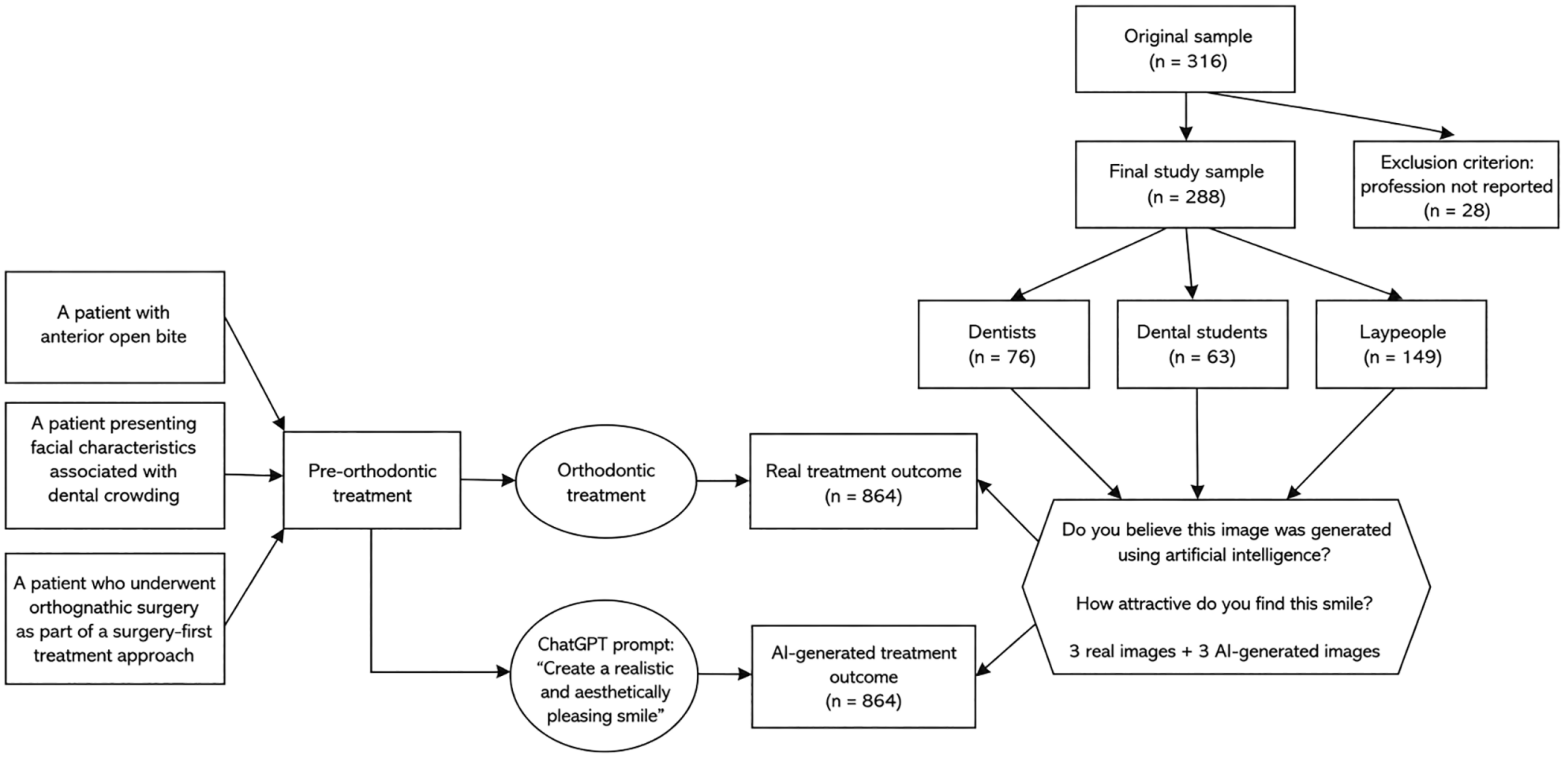
Study flow chart illustrating the methodology used to generate the real and AI‐generated facial images.

In addition to image evaluations, participants provided demographic data, including age, gender, educational level, employment status, marital status, and geographic region. Participants also identified their professional background to allow subgroup analyses based on expertise. The research flowchart is presented in Figure 1.

### 3.6 Pilot Study

A pilot study involving 40 participants (mean age = 32.6 years, SD = 8.4) was conducted to assess the test–retest reliability of the questionnaire. The sample included 12 dentists, 14 dental students, and 14 laypeople. Each participant completed the questionnaire twice, with a 20‐day interval between responses. The results indicated good test–retest reliability for the VAS scale, with an Intraclass Correlation Coefficient (ICC) of 0.82. The binary item assessing AI identification showed substantial agreement, with a Cohen's Kappa of 0.81 (*p* < 0.001). No significant differences were detected between the two time points for either variable (paired *t*‐test for VAS: t = 0.65, *p* = 0.52; McNemar's test for the binary variable: *p* = 0.41). Data from the pilot study were not included in the final analysis since participants could be identified via unique matching codes, whereas full anonymity was ensured for the main study sample.

### 3.7 Statistical Analysis

All responses were exported from Qualtrics and analysed using R software, version 4.4.0 (R Core Team 2025. R: A Language and Environment for Statistical Computing. R Foundation for Statistical Computing, Vienna, Austria). Descriptive statistics (frequencies, means, and standard deviations) were computed to summarize the characteristics of the sample and response patterns. To evaluate participants' performance in identifying AIGFO and RFO, we utilized the following metrics: accuracy, sensitivity, specificity, positive predictive value (PPV) and negative predictive value (NPV). A 95% confidence interval (CI) was determined for accuracy. Sensitivity was calculated to measure ability of correctly identifying AIGFO. Specificity was used to measure the ability of identifying RFO. AIGFOs were considered as positive cases and RFO as negative cases when calculating PPVs and NPVs. To compare ability to identify AIGFO and RFO, attractiveness and employability ratings across participant groups, the following statistical methods were applied: Pearson's Chi‐Square Test was used to assess associations between participant groups and their ability to correctly identify AIGFO and RFO images, as well as their employability ratings. Each participant group was tested on both identification accuracy and employability ratings; Cohen's Kappa Coefficient to evaluate inter‐rater agreement beyond chance; Tests for Homogeneity to compare classification accuracy across groups.; Kruskal‐Wallis Test, a non‐parametric alternative to ANOVA, to compare attractiveness ratings across participant groups; The Mann–Whitney U test used to compare attractiveness between AIGFO and RFO; To evaluate distribution of perceived attractiveness of AIGFO and RFO results were visualized with violin plots including individual data points. The plot was generated using ggplot2 package in R. To account for repeated measurements within participants, Generalized Estimating Equations (GEE) were applied, including age and sex as covariates in all adjusted models. Statistical significance threshold of *p* < 0.05 was used in all analyses.

## 4 Results

Of the 288 participants, 51.7% (*n* = 149) were laypeople, 26.4% (*n* = 76) practicing dentists, and 21.9% (*n* = 63) dental students. The mean age was 32.4 years (SD = 12.0), and most participants were female (63.8%). Participants represented all major regions of Brazil, with the majority residing in the Southern region (63.0%). (See S2). The descriptive results for the pre‐treatment, post‐treatment, and AI‐generated images are provided in S3.

Participants' classification performance varied by professional background (Table 1). Dentists correctly identified 35.4% of AIGFO and 71.8% of RFO, with 64.6% of AIGFO misclassified as real. Dental students showed a higher correct identification of AIGFO (47.5%) and the highest correct recognition of RFO (88.5%), with only 11.5% of RFO misclassified as AI‐generated. Lay participants correctly identified 33.9% of AIGFO and 82.1% of RFO, while 66.1% of AIGFO were misclassified as real. Significant differences among groups were observed for both AI‐generated (*p* = 0.005) and real image classification (*p* < 0.001).

**TABLE 1:** Confusion matrices for differentiating real and AI‐generated images, categorized by levels of clinical experience.

Reference	Group	AI (AIGFO)	Real (RFO)	*p*
Participant classification: AI (AIGFO)	Dentists	35.4% (75)	28.2% (57)	0.005
	Dental students	47.5% (84)	11.5% (18)	
	Laypeople	33.9% (145)	17.9% (69)	
Participant classification: Real (RFO)	Dentists	64.6% (137)	71.8% (145)	< 0.001
	Dental students	52.5% (93)	88.5% (139)	
	Laypeople	66.1% (283)	82.1% (316)	

Classification performance differed across professional groups. Dentists achieved an overall accuracy of 53.1% (95% CI: 48.2%–58.0%), with low sensitivity for identifying AIGFO (35.4%) and moderate specificity for recognizing RFO (71.8%). Their PPV and NPV were 56.8% and 51.4%, respectively. Dental students demonstrated the highest performance, with an accuracy of 66.8% (95% CI: 61.4%–71.8%), higher sensitivity (47.5%), and the greatest specificity (88.5%). They also showed the highest PPV (82.4%) and NPV (59.9%). Lay participants showed intermediate accuracy (56.7%; 95% CI: 53.2%–60.1%), the lowest sensitivity (33.9%), and relatively high specificity (82.1%), with PPV and NPV of 67.8% and 52.8%, respectively. (Table 2).

**TABLE 2:** Metrics of distinguishing real and AI‐created images categorized by levels of clinical experience.

Reference	ACCURACY (95% CI)	Sensitivity	Specificity	PPV	NPV
Dentists	0.531 (0.482–0.580)	0.354	0.718	0.568	0.514
Dental students	0.668 (0.614–0.718)	0.475	0.885	0.824	0.599
Laypeople	0.567 (0.532–0.601)	0.339	0.821	0.678	0.528

**FIGURE 2:**
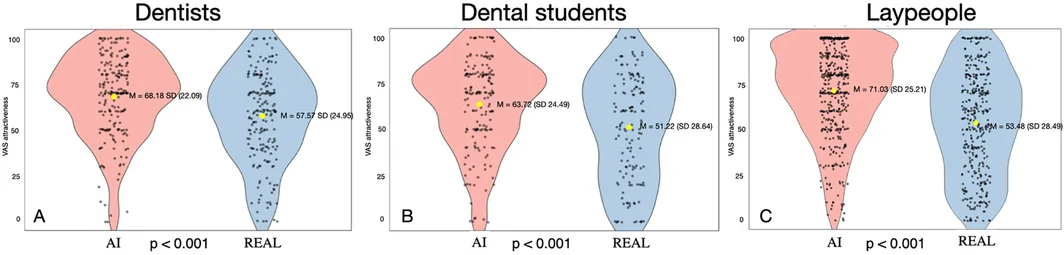
Dentist's evaluation of attractiveness using the VAS scale. Means are indicated by yellow dots (p‐value < 0.001). M = Mean, SD = Standard deviation.

**FIGURE 3:**
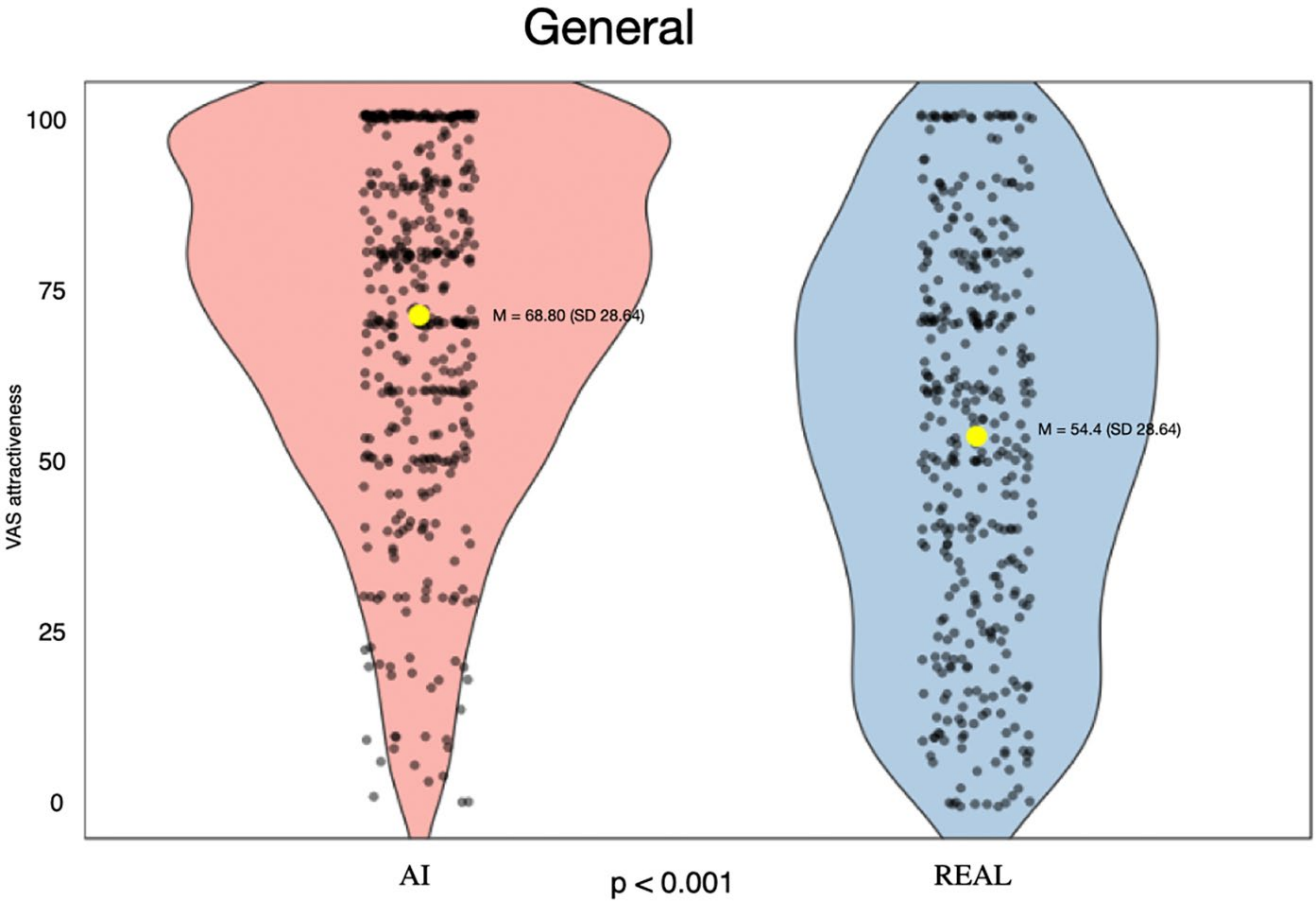
General evaluation of attractiveness using the VAS scale. Means are indicated by yellow dots (p‐value < 0.001). M = Mean, SD = Standard deviation.

Across all participants, the mean VAS attractiveness score was significantly higher for AIGFO (68.8) than for RFO (54.4) (*p* < 0.001). When stratified by professional background, the same pattern was observed. Dentists rated AIGFO at 68.2 versus 57.6 for RFO; dental students rated AIGFO at 63.7 versus 51.2 for RFO; and laypeople rated AIGFO at 71.0 versus 53.5 for RFO (Figure 2). The violin plots (Figures 2 and 3) demonstrate a concentration of responses within the central‐to‐upper range of the VAS scale for both image types, reflecting a central tendency in attractiveness judgments. A statistically significant difference in AIGFO ratings was observed among groups (*p* < 0.001), whereas no significant intergroup difference was found for RFO (*p* = 0.142) (Figure 3). Notably, lay participants exhibited greater dispersion in their ratings, while dentists demonstrated comparatively narrower distributions, indicating higher consistency in professional evaluations.

Regarding employability, Table 3 presents the participants' willingness to hire based on the evaluation of RFO versus AIGFO. The analysis revealed that participants were more likely to express willingness to hire individuals depicted in AIGFO compared to RFO. Specifically, 50.4% of participants indicated “Yes” for the AIGFO, whereas only 39.1% responded affirmatively for the RFO. Conversely, a higher proportion of participants selected “No” when evaluating RFO (15.5%) compared to AIGFO (6.88%). Additionally, 45.4% of participants reported that it was “Not possible to determine based on the image alone” for RFO, while a slightly lower proportion (42.8%) expressed the same for AIGFO. The difference between responses for real and AIGFO was statistically significant (*p* = 0.0071), indicating that image type had a measurable association on participants' hiring decisions. After adjustment for age and sex using GEE models, age showed a modest association with AI‐enhanced outcomes (OR = 1.02; 95% CI: 1.00–1.03), while sex was not significantly associated with misclassification, attractiveness ratings, or hiring decisions.

**TABLE 3:** Confusion matrices for participants' willingness to hire based on real vs. AI‐generated images.

	Real (RFO)	AI (AIGFO)
Hiring person	% (*n*)	% (*n*)
Yes	39.1% (285)	50.4% (410)
No	15.5% (113)	6.88% (56)
Not possible to determine based on image alone	45.4% (331)	42.8% (348)
Missing	135	50

*Note:* Missing: number of unanswered or skipped responses for the respective item.

## 5 Discussion

This study demonstrates that AI‐generated images were consistently perceived as more attractive and professionally appealing than real images. All participant groups struggled to accurately differentiate AI‐generated from real images. This trend, characterized by higher specificity than sensitivity, indicates that participants were generally more successful at identifying real images than distinguishing synthetic ones. These findings align with prior research highlighting the difficulty of detecting AI‐generated faces, even among trained observers [4, 12].

From an orthodontic perspective, these findings are highly relevant. Facial and dental aesthetics are well‐established determinants of social perception, influencing judgements of competence, trustworthiness, and employability [1, 2]. Orthodontic treatment aims not only to improve occlusal function but also to enhance facial harmony and smile aesthetics, which significantly impact psychosocial well‐being [14]. In the present study, AI‐generated outcomes were perceived as more attractive than real clinical results. This may reflect algorithmic optimization of features such as symmetry, smoothness, and proportional balance, which are consistently associated with perceived attractiveness [15, 16]. However, unlike orthodontic treatment, AI‐generated images are not constrained by anatomical, skeletal, periodontal, or functional limitations.

This discrepancy may contribute to unrealistic aesthetic expectations. Patient expectations are known to strongly influence satisfaction with orthodontic treatment outcomes [17]. If digitally optimized smiles that exceed biologically achievable results become widely disseminated, the gap between expected and realistic outcomes may widen, potentially affecting treatment demand, satisfaction, and clinician–patient communication. Furthermore, recent research shows that individuals often struggle to detect AI‐generated content across text, images, and multimedia formats [18, 19, 20]. As a result, the visual refinement inherent in AI‐generated images may override concerns about artificiality, favouring positive social judgements such as attractiveness and employability. Given the accessibility of generative tools, highly idealized facial images can be produced with minimal expertise, potentially reinforcing unattainable aesthetic standards and blurring the distinction between clinically achievable outcomes and digitally enhanced representations. In addition, the observed influence of age suggests that demographic characteristics may shape the perception and acceptance of AI‐generated orthodontic outcomes. Variations across age groups may reflect differences in digital exposure, expectations, and cognitive processing of facial features [21, 22]. Consistent with this, the GEE models showed that age had a modest but significant association with the evaluation of AI‐enhanced outcomes. Previous studies also indicate that age and gender may influence the perception of orofacial aesthetics and susceptibility to synthetic media [23, 24]. Although sex was not significant in the present study, demographic factors remain important in understanding responses to AI‐generated images.

Recent studies have demonstrated that synthetically generated faces are not only highly photorealistic but are also perceived as more trustworthy than real human faces [4]. These findings are consistent with previous evidence highlighting the remarkable realism achieved by AI‐generated facial images [25, 26]. Notably, this contrasts with earlier research suggesting that computer‐generated faces tend to be perceived as less trustworthy than real faces [27, 28, 29]. The present results reveal that AI‐generated orthodontic outcome images were rated not only as more attractive but also associated with higher employability judgements compared to real images in agreement with previous study [4]. This divergence from earlier findings suggests that the relationship between perceived authenticity, trustworthiness, and attractiveness is more nuanced than previously understood. A plausible explanation is that AI‐generated faces often embody enhanced averageness, smooth texture, and facial symmetry, attributes that are well‐documented predictors of increased aesthetic appeal and perceived trustworthiness [15, 16].

In a digital‐first society, where initial impressions are frequently mediated by profile pictures on social media and professional platforms, the impact of digitally optimized beauty on real‐world outcomes, such as hiring decisions and dating success, is increasingly salient [30, 31, 32]. The intersection of AI‐driven imagery and social media culture exacerbates the formation of unrealistic beauty standards. As users are exposed to a constant stream of hyper‐curated and synthetically perfected facial representations, perceptions of normative attractiveness are being recalibrated toward increasingly unattainable ideals [4, 33]. This phenomenon contributes to growing concerns about the psychological impacts of digital self‐presentation, including body dissatisfaction, diminished self‐esteem, and pressure to seek cosmetic interventions [30, 34].

Several limitations should be acknowledged. First, the cross‐sectional design does not allow causal inferences. Second, the study was conducted within a single national context, which may limit generalizability due to cultural differences in aesthetic perception. Third, although adjusted analyses using Generalized Estimating Equations (GEE) accounted for age and sex, stratified multivariable modelling by professional subgroup was not performed. Due to the limited number of outcome events within certain subgroup categories (e.g., specific combinations of professional background and categorical responses), stratified regression models would likely produce unstable estimates, inflated standard errors, and wide confidence intervals. Therefore, the GEE approach was utilized on the overall sample to ensure greater statistical stability while appropriately adjusting for confounders. Additionally, undergraduate dental students were analysed as a single group, as academic year was not recorded; differences in clinical training level may influence aesthetic perception and should be considered when interpreting subgroup findings. Furthermore, AI‐generated images were created using a single generative tool and a non‐optimized prompt, which may limit reproducibility and generalizability across other AI systems or prompting strategies. It should also be noted that the same image set was used in a separate study; however, the present investigation involved a distinct participant sample, different research questions, and independent outcome analyses, with no overlap in participants or duplicate reporting of results. From a clinical perspective, the findings suggest that AI‐generated orthodontic simulations may be perceived as more attractive than real treatment outcomes, potentially influencing patient expectations. The limited ability of dental professionals to consistently distinguish AI‐generated from real images makes clear the need for careful communication regarding achievable treatment results. Future research should explore different AI systems, refined prompting strategies, and the impact of AI‐generated visualizations on patient decision‐making in orthodontic practice.

## 6 Conclusion

Participants showed limited ability to accurately distinguish between real and AI‐generated images. AI‐generated orthodontic treatment outcome images are perceived as significantly more attractive and professionally favourable compared to real post‐treatment images. Across all participant groups, AI‐generated faces consistently received higher attractiveness ratings, with laypeople exhibiting the highest scores. Image origin significantly influenced employability judgements, with participants preferring AI‐images.

## Author Contributions

Otso Tirkkonen, Gil Guilherme Gasparello, Sergio Luiz Mota‐Júnior conceptualized and designed the study. Gil Guilherme Gasparello and Sergio Luiz Mota‐Júnior were responsible for the preparation and formatting of figures. Gil Guilherme Gasparello, Sergio Luiz Mota‐Júnior. Gil Guilherme Gasparello, Otso Tirkkonen Sergio Luiz Mota‐Júnior conducted the statistical analyses. Otso Tirkkonen, Marco Antonio Dias da Silva, and Felipe Calgaro Martins drafted the manuscript. All authors contributed to the translation and back‐translation process and critically reviewed and approved the final version of the manuscript.

## Funding

The authors have nothing to report.

## Ethics Statement

This study was approved by the Research Ethics Committee of the Pontifícia Universidade Católica do Paraná, Brazil, under approval number 6.861.105, CAAE: 77741824.5.0000.0020. The study was conducted in full accordance with the principles of the Declaration of Helsinki. Participation was voluntary, anonymous, and devoid of identifiable data. Informed consent was obtained from all participants and from individuals whose anonymized smile images were used, authorizing their use for research and educational purposes. The study complied fully with the Declaration of Helsinki and its amendments.

## Consent

Written informed consent was obtained from all individuals whose facial photographs were included in the study. Participants authorized the use of their images exclusively for research and survey evaluation purposes. All individuals provided informed consent to participate in the study. However, explicit authorization for publication of identifiable facial images in a scientific journal was obtained from only two of the three models. The third model, whose images included the open bite condition, consented to participate in the research but did not grant permission for image publication. In accordance with ethical guidelines and to ensure participant privacy, images from this model are not included in the Supporting Information.

## Conflicts of Interest

The authors declare no conflicts of interest.

## Supporting information


**Supporting Information S1:** presents the standardized frontal photographs used as survey stimuli, organized according to case/model and experimental condition, including pre‐treatment, real post‐treatment, and AI‐generated outcome images.
**Supporting Information S2:** provides the demographic and professional characteristics of the study population according to participant group.
**Supporting Information S3:** summarizes the descriptive outcomes according to image type and professional background, including AI‐recognition responses, positive hiring decisions, and VAS attractiveness scores.

## Data Availability

The data that support the findings of this study are available on request from the corresponding author. The data are not publicly available due to privacy or ethical restrictions.
