# Supplementary material for: Comparing Facial Attractiveness and Professional Acceptance Between Real and AI‐Generated Orthodontic Treatment Outcome Images
Source: Orthod Craniofac Res. 2026 May 18;29(5):963–71. doi: 10.1111/ocr.70144 (PMC13543177; doi:10.1111/ocr.70144)
Supplement: Supplementary file 1 [file OCR-29-963-s001.docx]

Supplementary material 1. Standardized frontal photographs used as survey stimuli. The figure presents representative facial images organized by model (top row: Model 1; crowding; bottom row: Model 2; surgery-first case) and experimental condition. Panels A and D depict the pre-orthodontic treatment condition; panels B and E represent the post-orthodontic treatment condition; and panels C and F show AI-generated outcomes.

All individuals provided informed consent to participate in the study. However, explicit authorization for publication of identifiable facial images in a scientific journal was obtained from only two of the three models. The third model, whose images included the open bite condition, consented to participate in the research but did not grant permission for image publication. In accordance with ethical guidelines and to ensure participant privacy, images from this model are not included in the supplementary material.

*Supplementary material 2. Characteristics of study population*

| **Variable name** | **% (n)** |  |  |
| --- | --- | --- | --- |
| **Education** | Dentist | Dental student | laypeople |
| High School Diploma or Equivalent | 0 % (0) | 63.5 % (40) | 39.6 % (59) |
| Undergraduate Degree | 11.8 % (9) | 30.2 % (19) | 31.5 % (47) |
| Postgraduate Degree (Specialization) | 32.9 % (25) | 3.2 % (2) | 22.1 % (33) |
| Postgraduate Degree (Master's or Doctorate) | 55.3 % (42) | 3.2 % (2) | 6.7 % (10) |
| Not Reported | 0 | 0 | 0 |
|  |  |  |  |
| **Sex (or gender)** |  |  |  |
| Male | 36.0 % (27) | 27.0 % (17) | 40.9 % (61) |
| Female | 64.0 % (48) | 73.0 % (46) | 59.1 % (88) |
| Missing | 1 | 0 | 0 |
|  |  |  |  |
| **Employment** |  |  |  |
| Retired | 0 % (0) | 0 % (0) | 2.7 % (4) |
| Self-employed or Entrepreneur | 56.6 % (43) | 1.6 % (1) | 25.5 % (38) |
| Unemployed or Temporarily Laid Off | 0 % (0) | 0 % (0) | 4.0 % (6) |
| Employed in a Permanent / Full-Time Position | 22.4 % (17) | 0 % (0) | 46.3 % (69) |
| Full-Time Student | 6.6 % (5) | 87.3 % (55) | 14.8 % (22) |
| Full-Time Student and Part-Time Employee | 9.2 % (7) | 11.1 % (7) | 3.4 % (5) |
| Other | 5.3 % (4) | 0 % (0) | 3.4 % (5) |
| Not Reported | 0 | 0 | 0 |
|  |  |  |  |
| **Marital status** |  |  |  |
| Married | 53.9 % (41) | 14.3 % (9) | 40.9 % (61) |
| Divorced or Separated | 3.9 % (3) | 1.6 % (1) | 5.4 % (8) |
| Single | 42.1 % (32) | 84.1 % (53) | 53.0 % (79) |
| Widowed | 0 % (0) | 0 % (0) | 0.7 % (1) |
| Not Reported | 0 | 0 | 0 |
|  |  |  |  |
| **Region** |  |  |  |
| Central-West | 0 % (0) | 3.2 % (2) | 16.8 % (25) |
| Northeast | 21.6 % (16) | 22.2 % (14) | 0.7 % (1) |
| North | 0 % (0) | 1.6 % (1) | 2.0 % (3) |
| Southeast | 31.1 (23) | 6.3 % (4) | 11.4 % (17) |
| South | 47.3 (35) | 66.7 % (42) | 69.1 % (103) |
| Not Reported | 2 | 0 | 0 |
|  |  |  |  |
| Age (mean(sd)) | 38.1 (11.0) | 23.5 (5.2) | 33.2 (12.4) |
| Not Reported |  |  |  |

**– VAS Attrtiveness Scores by Group and Image Type**

|  |  | Before | After | AI |
| --- | --- | --- | --- | --- |
|  |  |  |  |  |
| Dentist | Recognized image as AI generated (% (n)) | 26.6 % (54) | 28.2 % (57) | 35.4 % (75) |
|  | Positive hiring decision (% (n)) | 24.5 % (50) | 52.2 % (105) | 59.5 % (125) |
|  | VAS (mean (sd)) | 36.4 (24.0) | 57.57 (24.95) | 68.18 (22.09) |
|  |  |  |  |  |
| Dental student | Recognized image as AI generated (% (n)) | 7.5 % (12) | 11.5 % (18) | 47.5 % (84) |
|  | Positive hiring decision (% (n)) | 19.9 % (32) | 38.6 % (59) | 46.6 % (82) |
|  | VAS (mean (sd)) | 34.8 (26.8) | 51.22 (28.64) | 63.72 (24.49) |
|  |  |  |  |  |
| laypeople | Recognized image as AI generated (% (n)) | 16.7 % (67) | 17.9 % (69) | 33.9 % (145) |
|  | Positive hiring decision (% (n)) | 17.0 % (69) | 32.3 % (121) | 47.4 % (2039 |
|  | VAS (mean (sd)) | 37.0 (27.2) | 53.48 (28.49) | 71.03 (25.21) |

Supplementary Material 3. Descriptive outcomes (classification, attractiveness, and hiring decisions) according to image (Before Treatment, After treatment a AI generated) type and professional background.
